# Individualized neoantigen peptide immunization of a metastatic pancreatic cancer patient: a case report of combined tumor and liquid biopsy

**DOI:** 10.3389/fimmu.2024.1414737

**Published:** 2024-06-13

**Authors:** Tim Roehnisch, Mari Carmen Martos-Contreras, Mehdi Manoochehri, Mauro Nogueira, Franziska Bremm, Jan Dörrie, Jan Christoph, Meik Kunz, Wolfgang Schönharting

**Affiliations:** ^1^ Interdisziplinäres Onkologisches Zentrum (IOZ), Munich, Germany; ^2^ Precision Medicine and Cancer Research (PMCR GmbH), Karlsruhe, Germany; ^3^ Department of Dermatology, Friedrich-Alexander-Universität Erlangen-Nürnberg, Universitätsklinikum Erlangen, Erlangen, Germany; ^4^ Comprehensive Cancer Center Erlangen European Metropolitan Area of Nuremberg (CCC ER-EMN), Erlangen, Germany; ^5^ Deutsches Zentrum Immuntherapie (DZI), Erlangen, Germany; ^6^ Bavarian Cancer Research Center (BZKF), Erlangen, Germany; ^7^ Chair of Medical Informatics, Friedrich-Alexander University (FAU) of Erlangen-Nürnberg, Erlangen, Germany; ^8^ AG Bio-Medical Data Science, Martin-Luther-Universität Halle-Wittenberg, Halle, Germany

**Keywords:** pancreatic ductal adenocarcinoma, case report, neoantigen, immunotherapy, peptide immunization

## Abstract

This report details a case of pancreatic cancer with liver metastasis that exhibited a positive immune response to personalized immunization therapy. Our study involved the identification of neoantigens and their corresponding immunogenic peptides using an in-house bioinformatic pipeline. This process included the identification of somatic mutations through DNA/RNA sequencing of solid tumor tissue and blood liquid biopsy. Computational prediction techniques were then employed to identify novel epitopes, followed by the design and manufacture of patient-specific immunization peptides. In combination with standard-of-care chemotherapy, the patient received a sequence of 5 biweekly prime injections followed by 2 boost injections 2 and 5 months later. The peptides were emulsified in Montanide and the injection-site was conditioned with nivolumab and imiquimod. The combined regimen of peptide immunization and chemotherapy resulted in a notable decline in CA19-9 tumor marker levels following both prime and boost applications. Subsequent MRI assessments revealed a reduction in the size of liver metastases several months post-immunization initiation. Importantly, the patient showed and improved overall survival and reported an improved quality of life without experiencing significant treatment-related adverse effects. This case underscores the potential benefits of personalized peptide-based immunization as an adjunctive therapy in the treatment of advanced pancreatic cancer, showcasing promising outcomes in tumor marker reduction, tumor shrinkage, and enhanced patient well-being.

## Introduction

Pancreatic ductal adenocarcinoma (PDAC) remains a leading cause of cancer death worldwide with a 5-year overall survival of only 9% (1). There is a persistent increase in the incidence and minimal improvement in mortality rates, which will make PDAC the second leading cause of cancer-related mortality by 2030 (2). Combination chemotherapy is an important option for newly diagnosed patients with advanced and metastatic disease. However, almost all PDAC patients eventually relapse and second-line options are limited; therefore, alternative therapeutic options are warranted (3).

PDAC exhibits an immunologically “cold” tumor microenvironment (TME), therefore, immune monotherapies using checkpoint inhibitors in PDAC have little clinical efficacy (4). Individualized immunization strategies using therapeutic vaccines for specific patients or subgroups of patients based on tumor molecular profiling and the patient’s HLA-haplotypes may provide novel treatment options (5). Here, we report a pancreatic cancer case with liver metastasis which has benefited from the individualized peptide immunization.

## Case presentation

**Figure 1:**
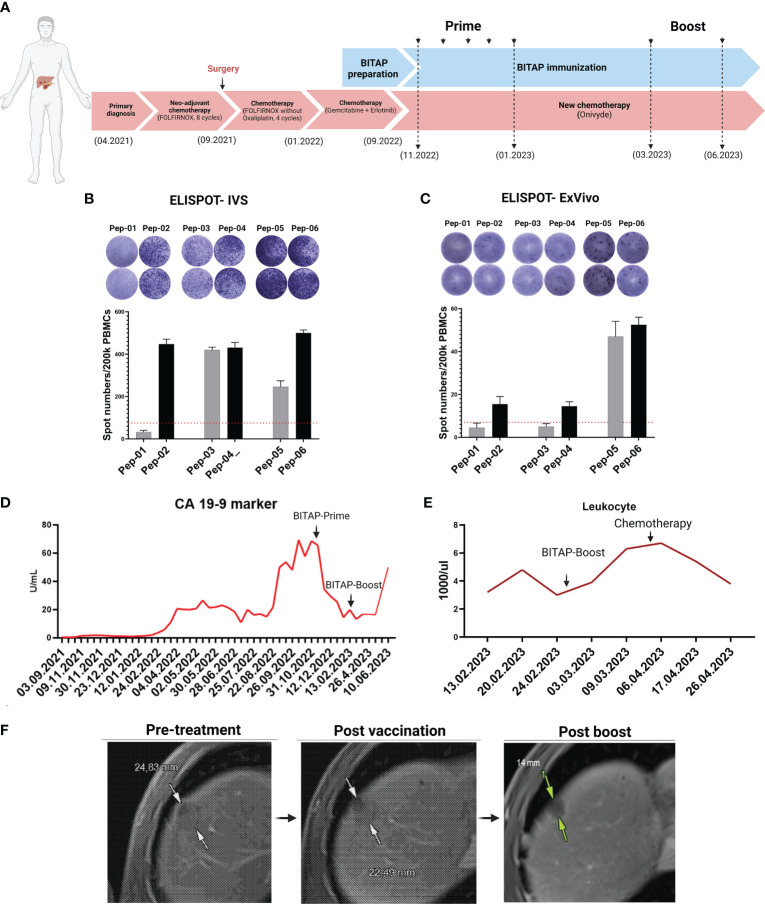
Treatment process and follow-up of metastatic pancreatic cancer patient through treatment periods. The patient received different treatments at different times. BITAP immunization was applied along with standard of care chemotherapy **(A)**. Immunogenicity testing of manufactured peptides by IVS- **(B)** and Ex-Vivo **(C)** IFN-γ ELISPOT assays. CA19-9 marker levels of the patient throughout the treatment **(D)**. Immuno-monitoring of the patient during the treatment **(E)** MRI scan was performed before and approximately three and six months after the start of BITAP immunization **(F)**.

A patient in their 50s was primarily diagnosed with pancreatic ductal adenocarcinoma on April 2021, and contrast medium sonography showed metastasic dissemination to the liver. The patient received 8 cycles of standard neo-adjuvant chemotherapy with FOLFIRNOX. In September 2021, the patient underwent surgery with total pancreatectomy and removal of four metastatic tumors from the liver. The pathological evaluation revealed moderately differentiated ductal adenocarcinoma with T2N0M1 stage. After surgery from September 2021 to January 2022, the patient received 4 cycles of adjuvant chemotherapy with FOLFIRNOX without Oxaliplatin. During the chemotherapy, CA19-9 level raised again, and CT scan showed 2 liver metastases. Therefore, new chemotherapy regimens were initiated, with Gemcitabine and Erlotinib administered from March 2022 to September 2022, followed by a switch to Onivyde starting in October 2022 (**Figure 1A**).

## Methods

### Next generation sequencing and preparation of immunization pool

Genomic DNA and RNA extracted from tumor tissue, along with genomic DNA and cell-free DNA (cfDNA) obtained from blood samples, were subjected to next-generation sequencing (NGS) analysis (**Supplementary Figure 1**). The selection of neoantigen-containing peptides in the immunization peptide pool was performed according to our in-house BioInformatic Tumor Address Peptides (BITAP) analysis pipeline and using sequencing data of tumor, blood and liquid biopsy (6). The peptides were then produced through chemical synthesis at >90% purity to generate the immunization peptide pool (Intavis Peptide Services GmbH, Tübingen, Germany). The details of the immunization peptide pool preparation are explained in the **Supplementary Materials**.

### Personalized peptide immunization

The BITAP peptide pool contains six synthetic peptides (600 µg/peptide) and XS15 adjuvant for the injections in every immunization cycle. On the bedside, the BITAP immunization peptide pool was emulsified in Montanide ISA 51 VG (Seppic), which then was applied subcutaneously (s.c.) on each date at 2-4 locations (left/right upper arms and/or abdomen). Twenty to thirty min before injection, 1300 μg nivolumab (Opdivo) was applied subcutaneously next to the injection site. After injection, 250 mg imiquimod (Aldara) was applied to the skin at the injection sites. In total, along with standard chemotherapy, five BITAP prime injections were administered every two weeks over the course of 2 months, from November 2022 to January 2023. Subsequently, two booster injections were given 2 and 5 months after the last prime injection.

### Immune response assays

The immunogenicity of each of the six peptides within the patient was assessed by measuring the number of specific IFNγ-secreting T cells in peripheral blood mononuclear cells (PBMCs) from the patient after the last priming injection. Enzyme linked immuno spot (ELISPOT) assays were performed using PBMCs that had been pre-stimulated with the peptide pool (IVS) and with ex vivo-isolated cells. As shown in **Figure 1B** and **Supplementary Figure 2A** the number of IFNγ positive spots indicated positive responses from the patient to 5 out of 6 peptides in the IVS assay against peptide concentrations as low as 20 nM. Ex vivo-detectable responses were observed against 4 out of 6 peptides (**Figure 1C** and **Supplementary Figure 2B**). Controls without peptide showed no IFNγ -producing cells (**Supplementary Figure 2**). Besides, the result shows that addition of delivery vector leads to an increase in the response rate (Peptides 2, 4 and 6 compared to peptides 1, 3 and 5, respectively) (**Supplementary Table 1**).

### Clinical outcome

This patient is a case study which benefits from peptide immunization treatment. After injections of prime and booster doses and in combination with chemotherapy, the patient’s follow-up showed beneficial results. As shown in **Figure 1D**, the application of BITAP immunotherapy in combination with new chemotherapy (Onivyde), resulted in a rapid decline in the levels of CA19-9 tumor marker during the prime application. Interestingly, the CA19-9 tumor marker again started to decline and stabilized after the first booster injection. During the treatment, the patient’s immune monitoring revealed a rise in the overall leukocyte count, whereas it decreased after chemotherapy (**Figure 1E**).

Additionally, the Magnetic Resonance Imaging (MRI) scans, which were performed before and approximately three months after immunotherapy, showed a regression of a liver lesion from 25 to 22 mm, as shown in **Figure 1F**. The subsequent MRI scan, conducted approximately six months after initiating immunization, revealed further regression of the liver lesion from 22 mm to 14 mm compared to the previous check-up.

During the follow-up visits, the patient felt a renewed sense of hope and optimism that had been lost during the previous stages of their chemo treatment; and unlike some of the harsh side effects they had experienced with chemotherapies, the BITAP injections didn’t lead the patient to feel the usual nausea or fatigue. Over time, as he continued with the BITAP immunization, the patient’s health status showed positive indications, and they began to notice gradual improvements in their overall well-being, increased energy levels with notable reductions in cancer-related symptoms. The patient exhibited a remarkable survival of 23 months post-surgery, including 10 months following the initiation of immunization. During this period, 9 months were characterized by a satisfactory quality of life attributable to the treatment regimen.

### Treatment-related adverse effects

The patient was actively encouraged to participate in their treatment journey by promptly documenting and reporting any new or unusual symptoms. Following injections, the patient experienced mild local reactions at the injection sites, including redness, swelling, and pain. A slight muscle shaking was also observed, but it occurred exclusively after the initial two injections. Additionally, the patient reported the occurrence of red granulomas at the injection sites days after injections, which later underwent a change in colour, turning darker, often to a bluish-black hue. No systemic symptoms or side effects were documented.

## Discussion

This case report provides valuable insights into the potential advantages of integrating neoantigen-based immunotherapy with standard chemotherapy in metastatic pancreatic ductal adenocarcinoma. The application of BITAP immunization, in combination with chemotherapy, demonstrated that individualized neoantigen peptide immunization is feasible, has tolerable adverse effects, and induces positive neoantigen-specific response in T cells. The observed decline in tumor markers, the immune response activation, the tumor regression documented by MRI, and the improved patient well-being collectively suggest a positive effect of BITAP immunization in combination with standard-of-cares SoCs. This finding aligns with emerging research indicating that combination strategies of chemo- and immunotherapies may yield more comprehensive and effective treatment outcomes (7, 8). In a recent phase I trial of an individualized neoantigen vaccine, a tolerable and neoantigen-specific T cell response was reported in 50% of the pancreatic cancer patients.

Furthermore, the immune monitoring data showing an increase in the overall leukocyte count during BITAP immunotherapy, which is consistent with immune activation. The observed decline in leukocyte count after chemotherapy, while expected due to the cytotoxic effects of chemotherapy on rapidly dividing cells, highlights the immune-suppressive nature of conventional treatments. While the initiation of new chemotherapy (Onivyde) coincided with the beginning of immunizations in our case study, we acknowledge the potential for confounding factors and the limitation of lacking a control group to definitively attribute observed outcomes solely to the new chemotherapy regimen.

The MRI findings show the regression of liver lesions and provide convincing evidence of the therapeutic efficacy of the combined approach. This reduction in lesion size supports the idea that multi-peptide immunotherapy in combination with chemotherapy, could exert a positive influence on tumor regression and control. These observations align with the concept of immunomodulation as a mechanism for enhancing the tumor microenvironment and facilitating anti-tumor immune responses (9). The patient’s positive health status and increased energy levels following the treatment further emphasize the potential benefits of the BITAP immunization and chemotherapy combination. These improvements emphasise the potential not only for increasing immune response, and improved clinical outcomes but also for enhanced quality of life for patients undergoing such treatment regimens.

## Conclusions

In this patient, it was observed that although administration of the peptide immunization did not result in complete remission, however it demonstrated a notable enhancement in the disease management and patients’ quality of life. This improvement was accompanied by favorable changes in tumor marker levels, indicating a positive therapeutic response without achieving complete eradication. These findings underscore the potential of the personalized immunization in inducing immune system and positively impacting the overall well-being of patients. Recruiting patients within a clinical trial is essential to validate the efficacy and safety of the BITAP immunization approach.

## Data availability statement

The original contributions presented in the study are included in the article/**Supplementary Materials**. Further inquiries can be directed to the corresponding author.

## Ethics statement

The requirement of ethical review and approval for the studies involving humans was waived. The studies were conducted in accordance with the local legislation and institutional requirements. The participants provided their written informed consent to participate in this study. Written informed consent was obtained from the individual(s) for the publication of any potentially identifiable images or data included in this article.

## Author contributions

TR: Conceptualization, Investigation, Methodology, Resources, Writing – review & editing. MM-C: Data curation, Investigation, Software, Validation, Writing – review & editing. MM: Conceptualization, Data curation, Formal analysis, Methodology, Project administration, Resources, Supervision, Validation, Visualization, Writing – original draft, Writing – review & editing. MN: Data curation, Investigation, Methodology, Writing – original draft, Writing – review & editing. FB: Investigation, Methodology, Visualization, Writing – original draft, Writing – review & editing. JD: Data curation, Investigation, Methodology, Visualization, Writing – original draft, Writing – review & editing. JC: Data curation, Writing – review & editing. MK: Data curation, Formal analysis, Writing – review & editing. WS: Conceptualization, Formal analysis, Investigation, Methodology, Supervision, Writing – review & editing.
