# Supplementary material for: Individualized neoantigen peptide immunization of a metastatic pancreatic cancer patient: a case report of combined tumor and liquid biopsy
Source: Front Immunol. 2024 Jun 13;15:1414737. doi: 10.3389/fimmu.2024.1414737 (PMC11208475; doi:10.3389/fimmu.2024.1414737)
Supplement: Supplementary file 1 [file DataSheet_1.pdf]

Supplementary Material

1 Supplementary Data

1. Material and methods

1.1 Next Generation Sequencing and Bioinformatics

Formalin-fixed paraffin-embedded (FFPE) tissue samples were used for whole exome and transcriptome sequencing. In that respect, after tissue sectioning and evaluation by pathologist, DNA and RNA were isolated from FFPE tumor blocks with at least 40% tumor cells (MagMax FFPE DNA/RNA Ultra; Thermo Fisher). DNA from PBMCs in peripheral blood was isolated (QIA Symphony DSP DNA Mini Kit 96, Qiagen) and considered as normal tissue. Additionally, for the liquid biopsy, three 10-mL peripheral blood samples were collected in Streck cell-free DNA tubes and used for cfDNA isolation (Suppl. Figure 1).

Library preparation for whole exome sequencing (WES) was performed using Twist Human Core Exome kit with RefSeq and Mitochondrial Panel (Twist Bioscience). Library preparation for RNA-seq was carried out by SMART-Seq Stranded Kit (Takara). The quality controls were performed by fluorescence-based quantification method and fragment length analysis. The DNA and RNA-seq libraries were sequenced on a NovaSeq 6000 using 2x100 bp paired end reads which resulted in about 320 and 94 million reads, respectively.

Bioinformatic analysis was performed by our in-house BITAP pipeline. BITAP workflow is a computational tool divided in two steps. Firstly, in the analytical process, BITAP infers the somatic tumor mutations from the Whole Exome Sequencing (WES) of tumor and blood samples. The row data will then be preprocessed, aligned and filtered (fastQC, skewer, BWA mem and samtools). The HLA typing, gene expression and gene fusions will be defined from bulk-tumor RNAseq. The somatic mutations are called and annotated with the VEP tool (McLaren et al., 2016). Gene fusion is obtained by star-fusion (Haas et al., 2019), ARRIBA (Uhrig et al., 2021) and annoFuse tools (Gaonkar et al., 2020). BITAP workflow calculate gene expression in TPM through the RNAseq analysis after the alignment with STAR and expression calculation with RSEM (Li & Dewey, 2011)

In the second step, which is the immune prediction step, The binding affinity is calculated for the peptides by MHCflurry and NetMHCIIpan (Reynisson et al., 2020) (O'Donnell et al., 2018, 2020) for HLA class I and class II, respectively. Physicochemical properties are calculated with Protparam, and finally the pMHC immunogenicity will be predicted by a machine learning model, and ranked according to our parameters.

1.2 Peptide design and manufacturing

The peptides were then produced through chemical synthesis at >90% purity to generate the immunization peptide pool together with XS15 as an adjuvant. The immunization pool contains six different peptides encompassing single nucleotide variation (SNV) neoantigens, which were manufactured in the form of long peptide both with and without a delivery vector (Supplementary table 1). The peptides include different epitopes; the single-epitope and multi-epitope peptides. The various epitopes within each peptide were connected using a standard cleavable linker (3-AA size).

1.3 Immunogenicity Testing using Interferon-γ ELISPOT assay

The T-cell responses to the peptides were monitored in peripheral blood mononuclear cells (PBMCs) isolated from blood drawn before vaccination (data not shown) and after the priming phase (Supplemental figure 2). The ELISPOT technology allows to determine the number of cells that produce a certain cytokine (here IFNγ) with high sensitivity. Such analyses can be performed directly ex vivo or after a pre-stimulation of the cells in vitro.

In Ex Vivo ELISPOT, immune cells are isolated directly from the individuals, typically peripheral blood, and are not cultured or stimulated in vitro prior to the assay. The isolated PBMCs are directly plated onto ELISPOT plates coated with capture antibodies. The cells are incubated in presence of the respective peptide, allowing any antigen-specific immune responses to be captured and detected by the ELISPOT assay (2). Ex vivo ELISPOT assays reflect the immune response present in the individual at the time of sample collection and thus provide information about the ongoing immune response. To increase the sensitivity of the assay, the cells can be prestimulated in vitro, resulting in an expansion of the specific T cells. This further increases the sensitivity, but reduces the accuracy of the assay.

The ELISPOT assays were performed as described elsewhere (1): to determine the frequencies of specific T cells in the blood of the patient, PBMCs were isolated from 50 ml of patient blood by lymphoprep (Axis-Shield PoC AS) density gradient centrifugation. The purified cells were either used directly (ex vivo) for the ELISPOT, or were in vitro stimulated (IVS) with a pool of the peptides at a concentration of 200 nM each in T-cell medium (1) in presence of IL-2 and IL-7 for 15 days. Anti-IFNγ-coated 96-well ELISPOT plates (Mabtech, Hamburg, Germany) were used according to manufacturer's instructions. PBMCs were seeded at a density of 2 × 105 per well, and stimulated with the individual peptides at concentrations of 2 µM, 200 nM, and 20 nM. After 20 h of incubation at 37°C, the ELISpot plates were washed and stained with horseradish peroxidase (HRP)-conjugated anti-IFNγ antibody. All tests were performed in duplicate and included negative (No peptide) and positive controls (20 ng/ml SEA and 5 µg/mL PHA-L). ELISpot assays were evaluated using the Zeiss ELISpot Reader and the KS ELISpot 4.13.0 software. The responses were considered positive when the numbers of IFN-γ–secreting cells were at least 2-fold above the negative control. The results revealed high immunogenicity of the peptides: After IVT, all peptides but peptide 1 showed strong responses, partially even exceeding the maximum detection limit of the assay. Of note is that the peptides were recognized even at low concentrations of 20 nM, indicating a high functional avidity of the T cells. (Supplemental figure 2A). Even without prestimulation, the ELISPOT showed detectable numbers of specific T cells ex vivo, with peptide 5 and 6 again showing high avidity responses.

2 Supplementary Figures and Tables

2.1 Supplementary Figures

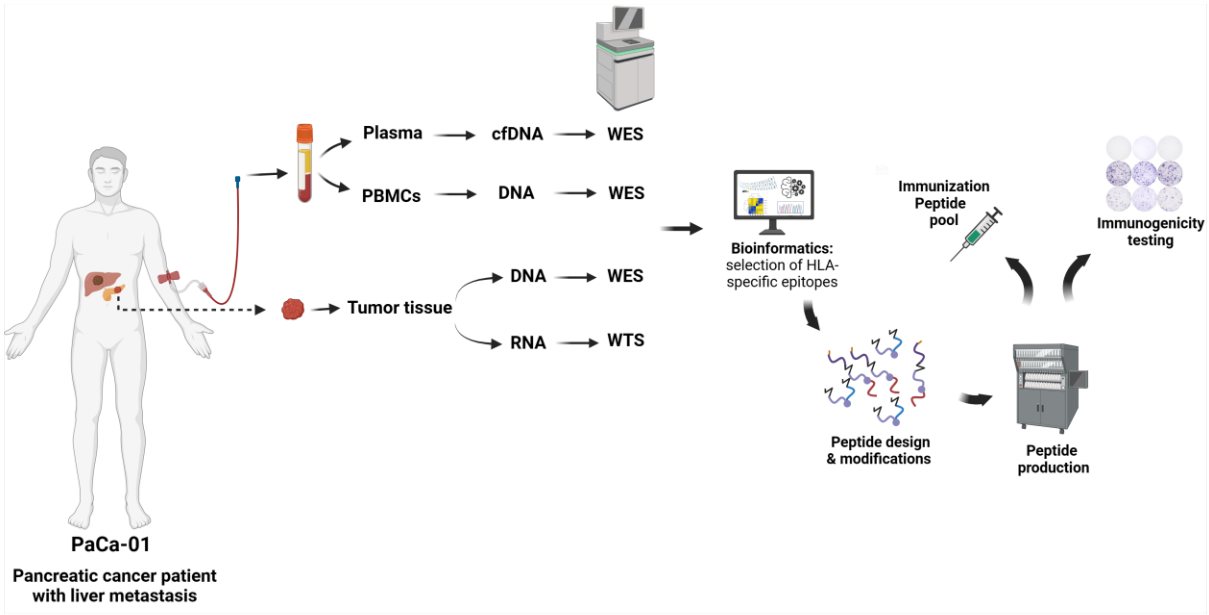

Supplementary Figure 1. Workflow of immunization peptide pool preparation from patient materials, and molecular analysis to the personalized immunogenic peptide pool.

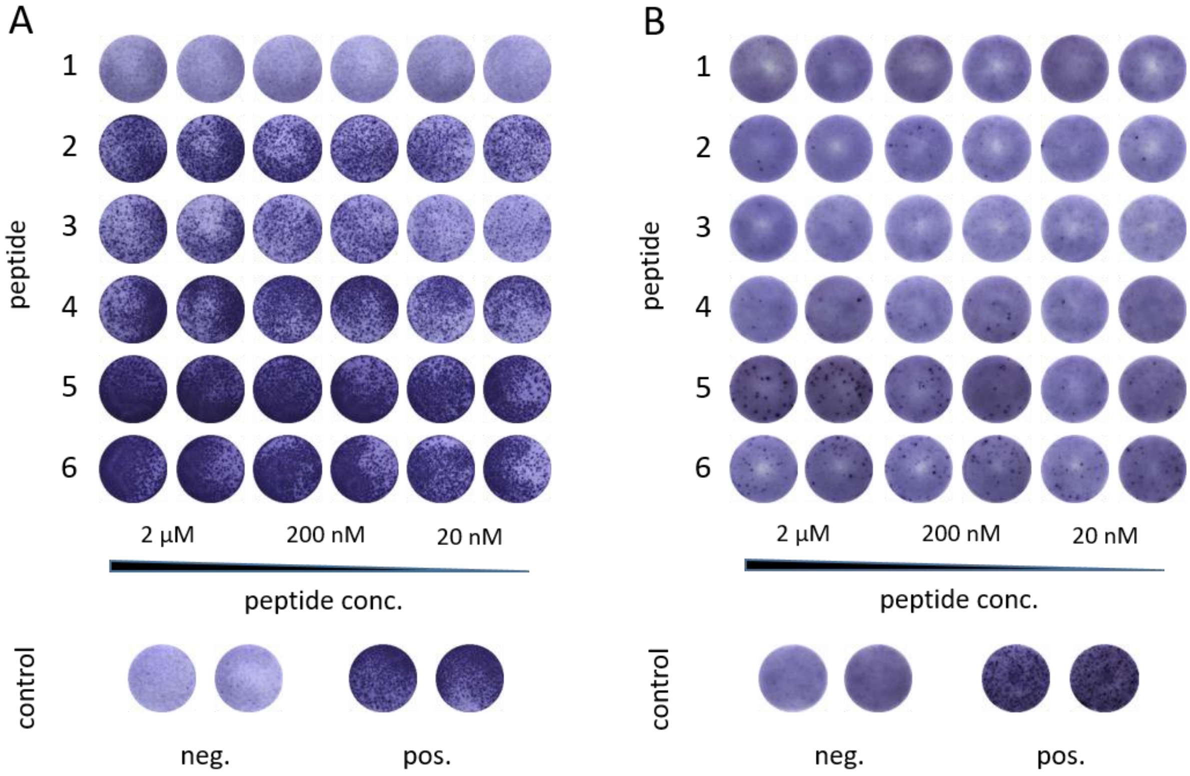

Supplementary figure 2: Peptide-specific immune responses after immunization. PBMCs were isolated after the priming phase of the treatment and were analysed for reactivity to the vaccination peptides depicted in Supplemental table 1. PBMCs were either prestimulated for 15 days with a pool of all peptides at 200 nM each (A) or were used directly ex vivo (B). In an IFNγ-ELISPOt-assay, 200.000 cells per well were stimulated with each individual peptide at concentrations of 2 µM, 200 nM and 20 nM in duplicate. Stimulations without peptide (neg.) and with SEA and PHA-L (pos.) served as controls.

Supplementary table 1: list of manufactured peptides (BITAP-1)

ID	Peptide	Gene/s	Antigen Type	AA change
Peptide-01	KLVVVGAVGVGKSALTI	KRAS	TSA: SNV	G12V
Peptide-02	KLVVVGAVGVGKSALTIRQIKIWFQNRRMKWKK	KRAS	TSA: SNV	G12V
Peptide-03	LANSPDLSHLAYYVTKVKEEDAF	ADAM8-NUP50	TSA: SNV	A399S-E351K
Peptide-04	LANSPDLSHLAYYVTKVKEEDAFRQIKIWFQNRRMKWKK	ADAM8-NUP50	TSA: SNV	A399S-E351K
Peptide-05	LQEFRRFQDPGRIVAIV	CEMIP2	TSA: SNV	L301R
Peptide-06	LQEFRRFQDPGRIVAIVRQIKIWFQNRRMKWKK	CEMIP2	TSA: SNV	L301R

References

1. Gerer KF, Erdmann M, Hadrup SR, Lyngaa R, Martin LM, Voll RE, et al. Preclinical evaluation of NF-κB-triggered dendritic cells expressing the viral oncogenic driver of Merkel cell carcinoma for therapeutic vaccination. Ther Adv Med Oncol. 2017 Jul;9(7):451–64.

2. Sedegah M. The Ex Vivo IFN-γ Enzyme-Linked Immunospot (ELISpot) Assay. Methods Mol Biol Clifton NJ. 2015;1325:197–205.
